# Effects of functional training on sprinting, jumping, and functional movement in athletes: A systematic review

**DOI:** 10.3389/fphys.2022.1045870

**Published:** 2022-11-30

**Authors:** Marrium Bashir, Kim Geok Soh, Shamsulariffin Samsudin, Saddam Akbar, Shengyao Luo, Jaka Sunardi

**Affiliations:** Department of Sports Studies, Faculty of Educational Studies, Universiti Putra Malaysia, Seri Kembangan, Malaysia; Faculty of Sport Science, Universitas Negeri Yogyakarta, Yogyakarta, Indonesia

**Keywords:** functional training, sprinting, jumping, functional movement skills, athletes

## Abstract

This systematic review aims to illuminate the effects of functional training on sprinting, jumping, and functional movements in athletes. A systematic search of electronic databases—that include PubMed, EBSCOhost (Sport Discus), SCOPUS, ProQuest, Google Scholar, and additional references—was carried out using keywords associated with functional training, jumping, sprinting, functional movement skills, and athletes, in compliance with the Preferred Reporting Items for Systematic Reviews and Meta-Analyses (PRISMA) statement criteria. The Physiotherapy Evidence Database (PEDro) scale was used to measure the methodological quality of the studies included in the systematic review. Results: From a total of 220 studies, 15 included ones met all eligibility criteria and were scored between 4-5 points—considered as“ moderate quality”—by the PEDro scale. Most studies recorded positive effects of functional training on athletes’ sprinting, jumping, and functional movement. In contrast, a small number of studies did not find any positive effects of functional training on sprinting, squat jump, vertical jump, and countermovement jump due to the short duration and frequency of the training, as well as the lack of additional exercises that come with the interventions. Furthermore, the reviewed studies reveal that there is limited research within the literature on 5, 15, 25, and 50 m sprinting, squat jump, quadrant jump, and functional movement in athletes. Conclusion: Although the length of training interventions varied across studies in this systematic review, functional training interventions were found to help improve athletes’ performance. The review reveals that training duration, intensity, and frequency are some critical variables that need to be taken into account when developing a successful functional training intervention for athletes. More studies are required to evaluate the influence of different accessible functional training durations on athletes’ sprinting, jumping performance, and functional movement. Finally, further research needs to be done to investigate the impacts of functional training on performance and movement skills of male and female athletes at all levels in other sports.

**Systematic Review Registration**: https://inplasy.com/inplasy-2022-5-0130/, identifier INPLASY202250130

## Introduction

The enhancement of athletes’ performance is defined by the proper improvement of the athletes’ sprinting (Keiner et al., 2014; Haugen et al., 2015; Bhardwaj and Kathayat, 2021), jumping (de Lacey et al., 2014), and movement skills (Kraus et al., 2014). Increasing athletic performance requires continuous development of speed and jump parameters (Akbar et al., 2022). An athlete’s running-based performance on their starting speed, acceleration, and top speed (Yildiz et al., 2019). Recently, the development of athletes’ performance has attracted a lot of attention to the FT method (Feito et al., 2018). Functional training (FT) has been defined that the goal of functional training is to enhance skills in sport (Stenger, 2018). The target movement is improved using this training method, which is often carried out by simulating the desired movement, rather than a specific muscle (Beckham and Haper, 2010). In the past decade, there has been a greater focus on the value of functional movement for both health and athletic performance (Barnett et al., 2021). Recent research indicates the importance of FT interventions for improving sprinting (Sander et al., 2013; Alonso-Fernández et al., 2017; Yildiz et al., 2019; Baron et al., 2020; Keiner et al., 2020; Usgu et al., 2020; Bhardwaj and Kathayat, 2021; Elvin Chacko Philip et al., 2022) and jumping performance (Baron et al., 2020; Kovac et al., 2022; Li, 2022). With such a broad description, research studies have different exercise programs with different designs (e.g., mini band exercises, Chop with squat position with plantar flex, plank exercises and core exercises) focuses have been included in the study on FT (Sander et al., 2013; Yildiz et al., 2019; Keiner et al., 2020). Additionally, numerous studies have found that functional training improves physical fitness in tennis players (Yildiz et al., 2019), handball players (Alonso-Fernández et al., 2017), soccer players (Abdel-Aziz Habib, 2018; Baron et al., 2020; Turna and Alp, 2020; Elvin Chacko Philip et al., 2022), netball players (Kovac et al., 2022), basketball players (Usgu et al., 2020; Bhardwaj and Kathayat, 2021), martial artists. Despite the fact that functional training is important for enhancing athletes’ performance, no publication has comprehensively discussed the effects of functional training protocols on sprinting, jumping and functional movement athletes.

A relatively recent technique developed for improving an individual running, leaping, and mobility abilities is functional training (FT) (Yildiz et al., 2019; Usgu et al., 2020; Kovac et al., 2022). FT is currently most clearly described as “purposeful learning,” but other definitions have stressed that its objective is to improve sports skills (Stenger, 2018). FT seeks to enhance a person’s movement patterns. As a result, FT is different from traditional training in the sense that it may involve exercises carried out to improve a particular movement (Pacheco et al., 2013). Ascertaining the difference among functional and traditional training programs can help to comprehend sustaining physical health and other well-being advantages (Weiss et al., 2010). Traditional exercise programs are thought to include exercises that isolate specific muscles to increase muscle strength more effectively (McGill et al., 2009). Based on this philosophy, the purpose of a traditional exercises is to develop a muscle or group of muscles’ strength or stamina, regardless of their muscle or muscle group movements during daily or sporting activities. Traditional, free-weight and machine-based training regimens that limit movement across a plane of motion (usually sagittal) may be less effective than activities that take place in more than one plane in daily life (Whitehurst et al., 2005).

According to numerous studies with strong evidence, most traditional training methods lack multi-articular and multiplanar components, which are essential for enhancing sports performance (Fernandez-Fernandez et al., 2016; Fernandez-Fernandez et al., 2020a; Fernandez- Fernandez et al., 2020b). If an athlete’s multi-joint movements, such as bending, turning, pushing, stepping and pulling are restricted or cannot perform in the sagittal, frontal, and transversal planes, this indicates that the athlete is functionally deficient, which will have an undesirable effect on their performance (Beckham and Haper, 2010; Ratamess, 2011). Because of this, the functional training program should be developed to mimic functional movements, or movements made during a person’s regular activities should be compatible with the exercises (Weiss et al., 2010). Conversely, according to several studies, FT combined multi-planar/multi-joint exercises, real-life activities, targeted injury prevention, and chain reactions inside the body prescribed *via* block periodization (Kasukawa et al., 2010; Stenger, 2018; Da Silva- Grigoletto et al., 2019). On the other hand, functional training (FT) has been introduced in sedentary women; the FT showed greater improvement in physical fitness parameters relevant to daily activities, with minimum important clinical differences (MCID) that were higher in magnitude and values than those of traditional training (de Resende-Neto et al., 2019). After 12 weeks of functional training, compared to intervention with functional exercises and traditional activities, older adults’ dynamic balance and agility improved by 7.6%, their jumping ability by 11%, and their lower limb strength by 15.3%, respectively (Resende Neto et al., 2018). Moreover, functional training is a new approach to exercise that has recently attracted a lot of interest in improving athletes’ physical fitness (Feito et al., 2018).

Functional movement skills have been characterized as physical movements with proper joint and muscle function and movement efficiency that reduces the risk of injury (Cook et al., 2006). However, the movement pattern that serves as the foundation for all other movements is referred to as functional movement skills (FMS) (Duncan et al., 2013), skills that are required for the successful engagement in health-improving physical activity (PA) and sports performance (Cook, 2003). Previous studies have attempted to explore the short-term relationships between FMS scores and a variety of fitness and athletic performance measures, including sprinting (Duncan et al., 2013; Girard et al., 2016; Silva et al., 2019) based on (Cook, 2003) model of movement development, where functional movement supports functional performance. The FMS test is a popular tool for assessing athletes’ quality of functional mobility (Bonazza et al., 2017). A deep overhead squat, hurdle step, in-line lunge, active straight leg lifts, shoulder mobility, trunk stability, and rotary stability were among the seven motions that made up the test (Shultz et al., 2013). Speed can be determined by timed 5, 10, or 30 m sprint tests, which are used to assess sprinting abilities. According to (Cook et al., 2006), propose an exercise program specifically created to improve the functional movement screen score to develop players’ functional capacity and treat movement disorders that have been identified.

Although FT interventions have been shown to improve performance outcomes, only a small number of research have focused on how FT training affects athletes. Recent publications include a number of systematic reviews of FT programs. However, previous review studies addressed the effect of FT on muscle strength, balance, mobility, activities of daily living in older adults (Liu et al., 2014), strength, endurance, balance (Wilke and Mohr, 2020), adult health (McLaughlin et al., 2020), physical fitness components (Xiao et al., 2021), and the risk of injury in athletes (Chen et al., 2021). To the best of our knowledge, no research has assessed how FT interventions affect athletes’ performance in terms of sprinting, jumping, and functional movements, which may enhance sports performance. Therefore, the purpose of the present review is to shed light on how functional training affects athletes’ ability to sprint, jump, and perform functional movements.

## Methods

### Protocol and registration

Preferred Reporting Items for Systematic Reviews and Meta-Analysis (PRISMA) criteria were followed in the current review (Moher et al., 2009), and this review was registered on the International Platform that can be accessed *via*: https://inplasy.com/inplasy-2022-5-0130/; INPLASY202250130.

### Eligibility criteria

According to the PICO the following criteria were used as a prior inclusion requirements for this systematic review: 1) research involving a control group or condition that could be used as a criterion for comparing an intervention; 2) studies that included athletes of all levels; 3) randomized control trial studies (RCTs; Randomized Control Trials); 4) studies written in the English language; and 5) studies that evaluated the effectiveness of functional movement skills like jumping and sprinting before and after an intervention.

Reviews and training-related investigations that do not concentrate on the effects of FT exercises were removed from the review category. The review also excluded studies in which athletes’ sprinting, jumping, and functional movement was not clearly described - related to injuries, psychology, and nutrition. Other exclusion criteria include participants were not athletes, no pre-post data, case reports and/or studies published in languages other than English, and studies for which only the abstract was published.

### Search strategy

From this systematic review, we searched the literature through PubMed, SCOPUS, ProQuest, EBSCOhost (SportDiscus), Google Scholar, and additional references through electronic databases from the inception of indexing until 7 April 2022. A comprehensive literature research, controlled vocabulary, and the opinions of experts were used to gather keywords (e.g., Medical Subject Headings: MeSH). The operators “AND” and “OR” were used in a Boolean search syntax. The words “functional movement screen,” “functional movement pattern,” “movement pattern,” “movement screen,” “functional movement skills,” “movement skills,” “players movement skills,” “athletes movement skills,” “functional movement performance,” “functional balance training,” “functional strength training,” “functional training,” “functional training exercises,” “functional exercises,” “jump,” “jumping,” “jump performance,” “jumping performance,” “sprinting,” “sprinting performance,” “speed,” “speed performance,” “sprint,” “sprint performance,” “athletes performance,” “players performance,” “female players,” “male players,” “athletes,” and players were used. Hand searches were done in addition to the main systematic electronic searches.

### Data collection and study selection process

**FIGURE 1:**
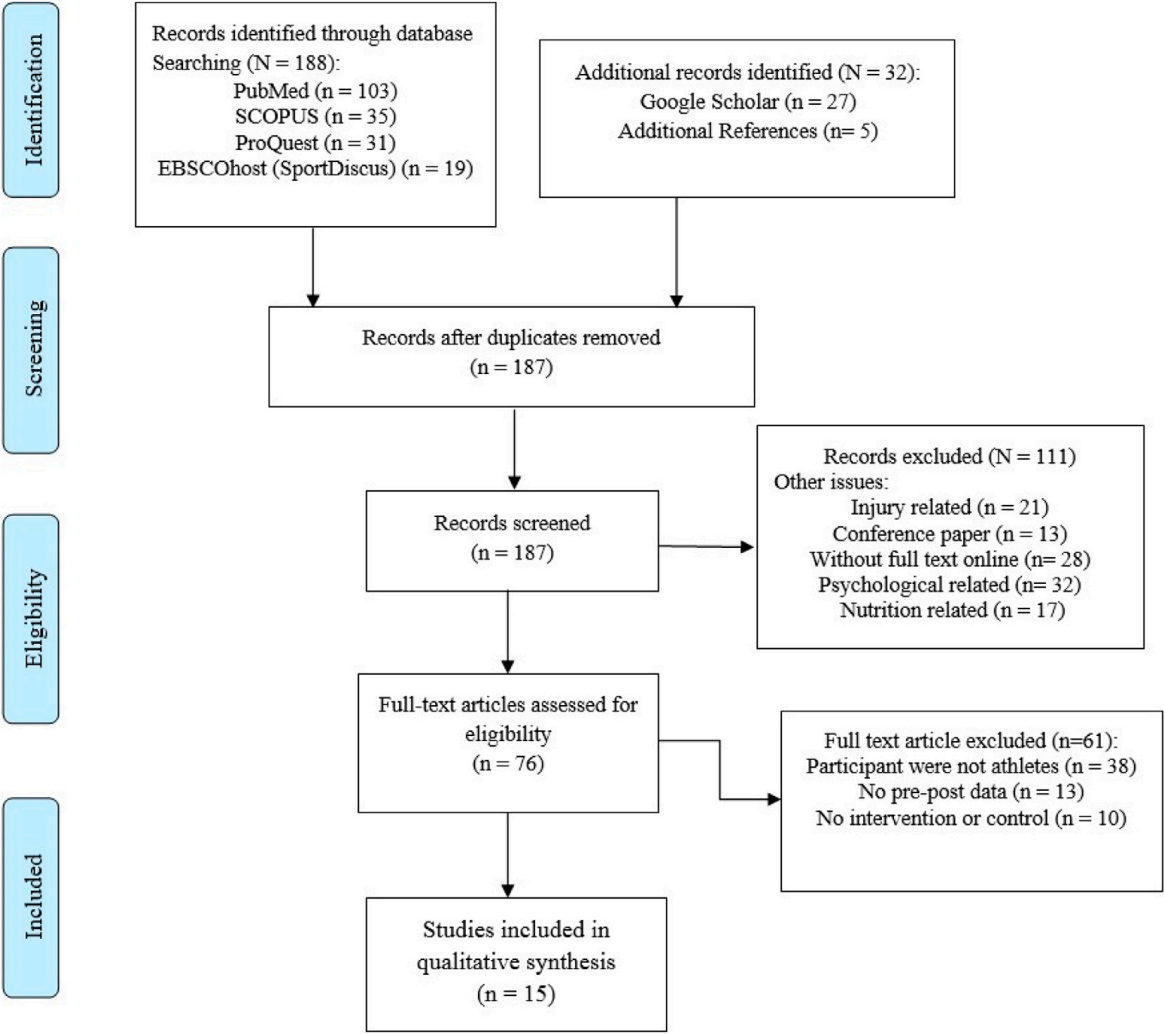
PRISMA flow chart of the study selection process.

As a first step, all appropriate article titles were reviewed to determine which studies should be included. Afterwards, the article abstracts were examined, and if deemed appropriate, the full-published papers were reviewed. The three authors (BM, KS, and AS) each contributed original work to the current study. The authors systematically extracted data from the included articles. Discussions with a third author helped to resolve any disagreements any two reviewers had regarding the study’s conditions. On top of that, full-text articles that did not meet the inclusion criteria were excluded (Figure 1).

### Data items and data extraction

For the current systematic review, jumping, sprinting and functional movement efficiency were selected as the primary outcomes in order to achieve a level of consistency throughout the studied investigations. The researchers looked at various jumping techniques and sprinting distances, which are regarded as measures of an athlete’s performing. The following details were included in the extracted data: study design and the number of participants per group. Additionally, sample demographic data such as age (years), weight (kg), height (m), level of fitness, training frequency (days/week), training lengths (weeks), and test results were also included in the retrieved data for FT characteristics.

### Study quality assessment

The Physiotherapy Evidence Database (PEDro) scale was used to assess the quality of the included studies in the systematic review. The PEDro checklist consists of 11 items with 10 being the highest score that could be given on the checklist—as item one is not counted into the total score. PEDro rates the internal study validity on a scale from 0 (high risk of bias) to 10 (low risk of bias). The study quality assessment was evaluated using the same 10-point scale as in various previous studies (Stojanović et al., 2017; Ramirez-Campillo et al., 2020), where three points represent poor quality, 4–5 points represent moderate quality, and 6–10 points represent high quality. The reviewers (BM, KS, and AS) followed this procedure and differences in study score were settled through internal discussions between them.

## Results

The results were examined and evaluated for studies that met the inclusion and exclusion criteria for the systematic review. This review includes fifteen studies that utilized RCTs to examine how functional training affected athletes’ ability in terms of their sprint performance, jump performance, and perform other functional movements. The studies were published from 2011 to 2022. Table 2 summarizes their main features. The study evaluation process is graphically schematized in Figure 1.

### Study selection

The flowchart displays the search for related articles (Figure 1). First, the search found a total of 220 articles from different databases (PubMed = 103, SCOPUS = 35, ProQuest = 31, EBSCOhost (Sport-Discus) = 19, google scholar = 27 and additional references = 5). In the initial screening, 187 studies were assessed after duplicates were eliminated. 76 studies were identified as potentially eligible during the initial screening. After the second screening, a total of 15 full text Publications were determined that fulfilled the eligibility requirements and were considered in the systematic review’s data synthesis.

### Study quality assessment

The 15 eligible studies achieved PEDro scores between 4-5, an indication of “moderate quality” (Table 1).

**TABLE 1:** Physiotherapy evidence database (PEDro) scale ratings^a^.

Study	Item 1	Item 2	Item 3	Item 4	Item 5	Item 6	Item 7	Item 8	Item 9	Item 10	Item 11	Total (from a possible maximal of 10)
Bhardwaj and Kathayat, (2021)	yes	yes	No	Yes	no	no	no	no	no	yes	Yes	5
Yildiz et al. (2019)	yes	yes	No	Yes	no	no	no	no	no	yes	Yes	4
Keiner et al. (2020)	yes	no	No	Yes	no	no	no	no	no	yes	yes	4
Tomljanović et al. (2011)	yes	yes	No	yes	no	no	no	no	no	yes	yes	5
Sander et al. (2013)	yes	no	No	yes	no	no	no	no	no	yes	yes	4
Usgu et al. (2020)	yes	yes	No	yes	no	no	no	no	no	yes	yes	5
Kovac et al. (2022)	yes	yes	No	yes	no	no	no	no	no	yes	yes	5
Baron et al. (2020)	yes	no	No	yes	no	no	no	no	no	yes	yes	4
Teixeira et al. (2020)	yes	yes	No	yes	no	no	no	no	no	yes	yes	5
Elbadry, (2014)	yes	no	No	yes	no	no	no	no	no	yes	yes	4
Alonso-Fernández et al. (2017)	yes	yes	No	yes	no	no	no	no	no	yes	yes	5
Turna and Alp, (2020)	yes	yes	No	yes	no	no	no	no	no	yes	yes	5
Elvin Chacko Philip et al. (2022)	yes	yes	No	yes	no	no	no	no	no	yes	yes	5
Abdel-Aziz Habib, (2018)	yes	no	No	yes	no	no	no	no	no	yes	yes	5
Li, (2022)	yes	no	no	yes	no	no	no	no	no	yes	yes	4

^a^ 1 indicates yes and 0 indicates no. Item 1, eligibility criteria specified; item 2, random allocation; item 3, concealed allocation; item 4, groups similar at baseline; item 5, participant blinding; item 6, therapist blinding; item 7, assessor blinding; item 8, fewer than 15% dropouts; item 9, intention-to-treat analysis; item 10, between-group statistical comparisons; item 11, point measures and variability data.

### Study characteristics

Table 2 displays the essential participant characteristics and the FT intervention’s programming parameters from the included studies. Five studies were conducted on male athletes (Tomljanović et al., 2011; Yildiz et al., 2019; Keiner et al., 2020; Elvin Chacko Philip et al., 2022), and three studies on female athletes (Elbadry, 2014; Alonso-Fernández et al., 2017; Kovac et al., 2022). Only one study featured a mix of males and females (Teixeira et al., 2020) while six did not disclose any gender information (Sander et al., 2013; Abdel-Aziz Habib, 2018; Baron et al., 2020; Turna and Alp, 2020; Usgu et al., 2020; Bhardwaj and Kathayat, 2021). A review of the fifteen studies revealed that the respondents’ ages ranged from 9.6 to 31.0 years (Table 2). Most studies reported information on respondents’ weight, height, and/or body mass index (BM). The subjects’ height and weight were recorded in ten studies (Sander et al., 2013; Yildiz et al., 2019; Keiner et al., 2020), the subjects’ BMI was mentioned in three studies (Alonso-Fernández et al., 2017; Baron et al., 2020; Teixeira et al., 2020), and four studies did not mention the subjects’ body weight, height, and/or BMI (Tomljanović et al., 2011; Bhardwaj and Kathayat, 2021; Elvin Chacko Philip et al., 2022; Li, 2022). The BMIs of participants in the studies were estimated from weight and height data. The subjects of the 15 studies had BMIs ranging from 17.28 to 25.4 kg/m^2^. Furthermore, of the fifteen articles, five studies (Sander et al., 2013; Elbadry, 2014; Alonso-Fernández et al., 2017; Yildiz et al., 2019; Keiner et al., 2020), revealed the training background of athletes, while four did not mention the training background of participants. The training histories of the athletes was reported in months to ensure continuity in the literature analysis. Moreover, across the reviewed studies, seven of them featured professional players, one study features moderately trained athletes, three studies examined adolescent athletes, one study featured elite youth athletes, two studies featured university level athletes and one study did not report this information. The subjects had an average training background of 36 months. The studies also featured a variety of different sports, which include football (*n* = 2), soccer (*n* = 5), basketball (*n* = 2), handball (*n* = 1), netball (*n* = 1), hammer throw (*n* = 1), tennis (*n* = 1), and gymnastics (*n* = 1). Overall, the included studies feature 85 subjects in seven control groups and 375 subjects in 21 different experimental groups (Table 2).

**TABLE 2:** Summary of details regarding each included study.

Study	Design	Sex	Study characteristics	Sports/Level of athletes	Training duration/Session duration (Mins)/Frequency	Main exercises	Training arrangement	Performance tests	Main outcomes
Bhardwaj and Kathayat, (2021)	Pre-post test	NR	FTG (*n* = 20) age 18–24	Basketball/Professional players	Length: 6-week/Time: NR min/Freq: 2 per week	Deep Squat, Hurdle step, Active Straight Leg Raise, Trunk Stability push up and Balance and Coordination	NR	Speed (50 m run test)	50 m↑
Yildiz et al. (2019)	Pre-post test	M	FTG (*n* = 10), TTG (*n* = 10), CG (*n* = 8) (mean ± SD; age: 9.6 ± 0.7 years; height: 134.1 ± 6.8 cm; body mass: 31.3 ± 4.1 kg; and training duration: 3.1 ± 1.3 years)	Tennis/ Professional prepubertal players	Length: 8-week/Time: 45 min/Freq: 3 per week	Control group: Forehand groundstroke ball feeding, Backhand groundstroke ball feeding, Smash, Service, Return Traditional training: Chest press, Shoulder press, Lateral pulldown, Triceps pushdown, Modified pushup, Sit-up Functional training: Squat, Squat, climbing man, Plank, Med. Ball throw, push up, Pull up	Control group: 1 min, 3 set, 1:1 rest, 60–100% Intensity Traditional training: 10 Reps, 3 Sets, 1:2 Rest Functional training: 16–20 rep, 1 set, 30 s, 50%–70% (ex.-iso-con.)	Speed, Jump (CMJ), FMS (observation- based test)	Speed↔, Jump↔, FMS score↑
Keiner et al. (2020)	Pre-post test	M	(STG: *n* = 11; PSTG: *n* = 11; FTG: *n* = 14; and CG: *n* = 12) (age: 17.45 ± 0.52 years old; height: 1.78 ± 0.06 m; and body mass: 73.0 ± 7.0 kg; mean and SD).	Soccer/Elite adolescent players	Length: 10 months/Time: 60 min/Freq: 2 per week	Vertical (e.g., squat, countermovement or drop jumps) and linear sprints, resisted sprints), horizontal (e.g., broad jump, triple jump) and change of direction sprints, Sprint and jump exercises,	(10-m to 30-m, up to 15 m with different degrees of change of directions), 3 sets, 6–8 repetitions	linear Sprint (20 m) Squat Jump (SJ)	20-m LS↔, SJ ↔
Tomljanović et al. (2011)	Pre-post test	M	FTG (*n* = 12), TRTG (*n* = 11) age 22–25	NR/Moderately trained athlete	Length: 5 weeks/Time: NR min/Freq: 3 per week	One-leg TRX-squats, TRX suspension rowing, TRX push-ups, Power-wheel leg flexion, Flow-in lunges, One-leg good morning	10–15 rep, load (80% of 1RM),	Jump (CMJ: AT, PEAKPWR, JH, GCT), speed (10 m, 20 m, 10–20 m),	JH↑, PEAKPWR↑, GCT↑, AT↔, 5– 10–5↔, 10 m↔, 20 m↔, 10–20 m↔
Sander et al. (2013)	Pre-post test	NR	NWPG (*n* = 65), WPSG (*n* = 56) age = 15.1 years; height = 170.9 cm; and mass = 62.3 kg	Soccer/ Elite youth players	Length: 8 days/Time: NR/ Freq: NR	Prone kneeling, Forearm bridging, bridging one leg to lift the pelvis, Lateral bridging with alternating leg flexion and leg extension, Crunches, Bridging both legs with alternate routes of the legs, Lateral bridging with hip abduction (908 knee angle),	8 rep, 6 times	Speed (linear sprint: 5 m, 10 m, 15 m, 20 m, 25 m, 30 m, and CDS:5 m left and right, 10 m left	5 m↑, 10 m↑, 15 m↑, 20 m↑, 25 m↑, 30 m↑, 5 m left↔, 5 m right↑, 10 m left↑, 5 m right↑
Usgu et al. (2020)	Pre-post test	NR	FTG (*n* = 14, average age: 26.6 ± 5.9 years), CG (n = 14, average age: 22.4 ±4.2 years)	Basketball/ Professional players	Length: 20 weeks/Time 75–85 min/Freq: 2 per week	Traditional Training: Bench Press, Shoulder Press, Box Jump, Leg Curl, Rowing Functional training: Push-Up, Abdominal Crunches, Jack Knife, Hip bridge, Russian Twist, Planks	Traditional Training: 8–10 rep, 50%–70% Intensity, Functional training: 8–15 rep, 3 sets	Speed (20 m), vertical jump (CMJ)	20 m↑, CMJ↑
Kovac et al. (2022)	Pre-post test	F	FTG (*n* = 12) (mean ± SD) age years 20.0 ± 1.5, Height (cm) 173.3 ± 6.3, Weight (kg) 69.3 ±7.4; CG (*n* = 19) age years 19.8 ± 1.5, Height (cm) 175.6 ±6.7, Weight (kg) 70.5 ± 8.1	Netball/Elite University players	Length: 6 weeks/Time: 30–40 min/Freq: 3 per week	Leg lowering, Hip flexor stretch, Leg lowering Leg, lock bridge, Deadlift patterning, Leg lowering Straight leg bridge—4 sets, 6 repetitions, Single-leg deadlift patterning RNT, Quadruped core activation, Plank with knee flexion, Rolling pattern, Elevated push-up	1–4 sets, 6 reps, 4 sets, 6 rep, 10 s hold, 2–4 sets, 6 reps, 4 sets, 6 reps, —4 sets, 6 reps, 2–4 sets, 6 reps, 4 sets, 6 reps, sets, 6 reps, 4 sets, 6 reps, 4 sets, 6 reps, 4 sets, 6 reps	Functional performance (FMS score)	FMS score↑
Baron et al. (2020)	Pre-post test	NR	FTG (*n* = 20) age years 17, Height (cm) 176, Body weight (kg) 64, BMI (kg/m2) 21.62, FAT (%)12.6 2.2	Football/ Professional players	Length: 12 weeks/Time: 45–60 min/Freq: NR	Back Stretching exercises, Mobilization of shoulder complex, Mobility of the thoracic spine, Extension of the thoracic spine; Four—point kneeling position, Thoracic spine rotation, Hip mobilization in the direction of flexion, extension, external and internal rotation, Ankle Mobilization towards the dorsiflexion, Central stabilization, stabilization of the ilio-lumbar-pelvic complex, Exercises with bands, global patterns, Balance, and coordination exercises,	(2–3 sets.)	Speed (5, 10, and 30 m) FMS (observation-based test)	5,10, and30 m↑ FMS↑
Teixeira et al. (2020)	Pre-post test	M/F	HTVF (*n* = 17) (9 F & 8 M; age: 31.0 ± 6.3 years; height: 168.8 ± 8.1 cm, body weight 73.6 ± 11.9 kg; BMI: 25.96 kg/m2) MTVF (*n* = 14) (8 F & 6 M; age: 26.6 ± 4.7 years; height: 167.2 ± cm, body weight: 75.8 ± 18.0 kg; BMI 27.33 kg/m2)	Training Center (Gymnastics, strength)/ NR	Length: 6 weeks/Time: 40–60 min/Freq: NR	Squats, bench-press, deadlift and their variations: handstands, bar exercises, ring, among others: handstands, bar exercises, ring	NR	Countermovement vertical jump height, Speed (20-m Sprint)	CMVJH test ↔, 20 m ↔
Elbadry, (2014); Alonso-Fernández et al. (2017)	Pre-post test	F	TG (*n* = 10), Age years 18.33 ± 0.5, Weight [kg] 69 ± 2.9, Height [cm] 167 ± 2.95, CG (*n* = 10), Age years 18.29 ± 0.8, Weight [kg] 68 ± 3.1, Height [cm] 168 ± 3.11	Hammer Throw/ University level	Length: 8 weeks/Time: 60 min/Freq: 3 per week	Vertical Jump Test (VJ), Seated Medicine Ball Throw (SMBT), leg strength (LS) back strength (BS) by the dynamometer, Dynamic strength test (DST)	NR	Vertical Jump Test,	Vertical Jump Test↑
Alonso- Fernández et al. (2017)	Pre-post test	F	GE (*n* = 7) Height (m) 1.64 ± 0.05, Weight (Kg) 63.17 ± 9.44, BMI (kg) 23.83 ± 3.46, % fat 30.13 ±4.16 GC (*n* = 7) Height (m) 1.66 ± 9.24, Weight (Kg) 67.29 ± 0.03, BMI (kg) 24.61 ± 3.93, % fat 32.00 ± 5.24	Handball/Professional players	Length: 8 weeks/Time: 10 min/Freq: 2 per week	Squat and jump, Dynamic split with both legs, Mountain Climbers, Coordination exercise on ladder, Plyometric jumps, Squat, Upper- body plyometric exercise, Coordination exercise on ladder	4 Reps, 2 Sets	Jumping (CMJ),	CMJ ↔
Turna and Alp, (2020)	Pre-post test	NR	FTG (*n* = 10) age years 25.2 ± 3.36, mean height 181.8 ± 4.44, mean weight 78.7 ± 5.96, mean sport age 6.40 ± 3.09: TTG (10), mean age years 22.90 ± 2.02, mean Height cm180.9 ± 6.06, mean weight kg 76.80 ± 6.44, mean sports age 4.40 ± 2.71.	Soccer/Professional players	Length: 8 weeks/Time: 60 min/Freq: 5 per week	FTG: Sit Ups, Medicine Ball Slams, Deadlifts, Bench Press, Sprints, Back Squats, Box Jumps, Lateral Lunge with Overhead Press, lunge, Battle Rope Waves, Walking Plank, Burpee, Squat Chop, Squat Lift, Curl Up Med Ball Throw, Squat with medicine ball Slam. TTG: Lactic elimination training, dynamic flexibility training, Speed training, tactical and shooting training	FTG: 3–5 sets, TTG: 5 min, 15 min, 20 min	Vertical Jump Test, 30 m Sprint Test	Vertical Jump test↔, 30 m Sprint test↔
Elvin Chacko Philip et al. (2022)	Pre-post test	M	FTG (*n* = 15), CG (n-15) age 17–25 N/A	Soccer/Recreational players	Length: 8 weeks/Time: 60 min/Freq: 2 per week	Push up with stability ball, one leg bench squat, Oblique bridge, single leg deadlift, single leg split squat, Planks, step up with heel raise	10 Reps, 3 Sets	Speed (30 m sprint)	30 m sprint↑
Abdel-Aziz Habib, (2018)	Pre-post test	NR	FTG (*n* = 15) (aged 17.54 ±0.7 years, body mass: 67.84 kg, height: 171.53 cm)	Soccer/Junior players	Length: 10 weeks/Time: 120 min/Freq: 3 per week	Functional resistance drills by using elastic bands: Chest Press & Single Leg Chest Press, Chest Fly, Back Row, Deltoid Fly, Triceps Press, High Biceps, Single Leg Balance Squat, Hamstring Curl, High Back Extension, High Torso Rotation, Kneeling Rollout, Leg Raise,	Reps 8–12, 3–5 sets	Quadrant jump test	Quadrant jump test↑

Abbreviation: ↑, Significant within-group improvement from pre-test to post-test; ↔ Non-significant within-group change from pre-test to post-test; N/A, Not available; M, Male; F, Female; FTG, Functional training group; STG, Strength training group; TRTG, Traditional resistance training group; NWPG, Normal soccer warm-up group; WPSG, Warm-up program supplemented group; HTVF, (High training-volume and frequency; MTVF, moderate training volume and frequency; TTG, Traditional training group; CG, Control group; BMI, Body mass index; m, Meter; kg, Kilogram; cm, Centimeter; mins, Minutes; wk, Week; LS, Linear sprint; SJ, Squat jump; JH, Jump height; AT, Air time: GCT, Ground contact time; CDS: PEAKPWR, peak power; CMJ, Counter movement jump; CMVJH, Countermovement jump height; FMS, Functional movement skills.

### Intervention characteristics

Three elements were used to report the intervention characteristics from the 15 eligible studies, namely intervention length, training duration, and training frequency. Intervention length: the shortest intervention length reported is 8 days (reported in one study) (Sander et al., 2013); one study reported a 5-week intervention length (Tomljanović et al., 2011); three studies reported 6-week intervention lengths (Teixeira et al., 2020; Bhardwaj and Kathayat, 2021; Kovac et al., 2022); six studies reported 8-week intervention lengths (Elbadry, 2014; Alonso-Fernández et al., 2017; Yildiz et al., 2019; Turna and Alp, 2020; Elvin Chacko Philip et al., 2022); one study reported a 12-week intervention length (Baron et al., 2020); one study reported a 20-week intervention length (Usgu et al., 2020), and one study reported 10 months intervention length (the longest) (Keiner et al., 2020). Training duration: four studies did not report the duration of intervention (Tomljanović et al., 2011; Sander et al., 2013; Bhardwaj and Kathayat, 2021; Li, 2022) while the rest did—the duration of training sessions ranged from a minimum of 10 min (Alonso-Fernández et al., 2017) to a maximum of 120 min (Abdel-Aziz Habib, 2018). Training frequency: studies reported that the frequency of exercise ranges from a minimum of 2 days per week (Alonso-Fernández et al., 2017; Keiner et al., 2020; Usgu et al., 2020; Bhardwaj and Kathayat, 2021; Elvin Chacko Philip et al., 2022) to a maximum 5 days per week (Turna and Alp, 2020). Four studies did not report the frequency of training (Sander et al., 2013; Baron et al., 2020; Teixeira et al., 2020; Li, 2022). Training intensity: Out of 15 including studies, only two studies reported training intensity 80% (Tomljanović et al., 2011) and 50%–70% (Yildiz et al., 2019).

## Outcomes

### Effects of FT on sprinting performance

Of the 15 included studies, ten provided data for sprinting performance. Two studies reported data on 5-m sprint (Tomljanović et al., 2011; Baron et al., 2020), Four studies reported data on 10-m sprint (Tomljanović et al., 2011; Sander et al., 2013; Yildiz et al., 2019; Baron et al., 2020), one study reported data on 15-m sprint (Sander et al., 2013), five studies reported data on 20-m sprint (Tomljanović et al., 2011; Sander et al., 2013; Keiner et al., 2020; Teixeira et al., 2020; Usgu et al., 2020), and only one study provided data on 25-m sprint (Sander et al., 2013). Meanwhile, four studies reported data 30-m linear sprint (Sander et al., 2013; Baron et al., 2020; Turna and Alp, 2020; Elvin Chacko Philip et al., 2022), and only one study reported data on 50-m linear sprint performance (Bhardwaj and Kathayat, 2021).

One study reported a mean difference of 0.95 s in 50 m sprint between the pre and post-test groups that indicates a positive effect of 6-week FTs on speed (Bhardwaj and Kathayat, 2021). However, another study showed that after 6 weeks, sprinting performance did not improve in any group [(F (1,29) = 1.014; *p* = 0.322)] (Teixeira et al., 2020). After 8-week, 3 days/week with 50%–70% intensity intervention, when the speed acceleration data were compared, neither the control group (CG), the traditional training group (TTG), nor the functional training group (FTG) showed any substantial differences between one another (*p* < 0.05). However, the difference between CG and FTG was statistically significant (*p* < 0.05) (Yildiz et al., 2019). According to another study, traditional strength training significantly increased performance of the linear sprint (LS) group compared to sprint and jump training, however, for FT (g 5 0.86–1.39), performance over time did not improve (Keiner et al., 2020). After a 5-week intervention with 80% intensity, post-test FT showed that it did not positively affect 10m, 20m, and 10–20 m sprints compared to the traditional resistance training group (Tomljanović et al., 2011). According to the study, FT is different from traditional training in the sense that it may involve exercises carried out to improve a particular movement (Pacheco et al., 2013).

Additionally, after implementing functional training for 12 weeks, the experimental group in a study consisting of football players did not experience any enhancement in acceleration within the range of 1.001 for a 5-m linear sprint (Baron et al., 2020). However, after the FT intervention, there was an increase in the following two variables: velocity and acceleration. The footballers’ acceleration and speed between 5m to 10m and 10m–30 m both recorded significant improvements (demonstrated while covering a considerable distance), amounting to 0.02 s (2.4 percent) and 0.04 s, respectively (1.5 percent) (Baron et al., 2020). There was a significant favor FT (*p* < 0.05) for an improvement in 20-m sprint performance when the pre- and post-test results were compared within groups (Usgu et al., 2020). Results showed that sprinting performance for 30 m were significantly improved between pre- and post-test values (Turna and Alp, 2020; Elvin Chacko Philip et al., 2022). However, according to one study, an FT program with warming up did not show significant results on sprints performance in the 30, 25, 20, 15, 10, and 5 m sprint (Sander et al., 2013).

### Effect of FT on jumping performance

Of the included studies, nine of them presented data for jumping, which include 14 training intervention groups. Four studies reported data on vertical jump (VJ) (Elbadry, 2014; Teixeira et al., 2020; Turna and Alp, 2020; Usgu et al., 2020), three studies reported data on countermovement jump (CMJ) (Tomljanović et al., 2011; Alonso-Fernández et al., 2017; Yildiz et al., 2019), one study reported data on squat jump (SJ) (Keiner et al., 2020), and one reported data on quadrant jump (QJ) test performance (Abdel-Aziz Habib, 2018). Group comparisons of the pre- and post-test results revealed a substantial improvement in FT Group’s (FTG) VJ performance (*p* 0.05) (Usgu et al., 2020). After 6 weeks, group’s performance for VJ height increased [(F (1,29) = 6.081; *p* = 0.050] (Teixeira et al., 2020). Between pre- and post-test values for VJ, there are statistically significant variations (Turna and Alp, 2020). In contrast, another study found no substantial difference in the VJ test results between the experimental and control groups (Elbadry, 2014).

The CMJ performances of control group (CG) and traditional training group (TTG) differed significantly from one another (*p* < 0.05). However, the differences between CG and FTG, and between TTG and FTG were more obvious (*p*, 0.001 and *p*, 0.01, respectively) (Yildiz et al., 2019). After 5-week, 3 days/week with 80% intensity of FT and traditional resistance training (TRT) were influenced differently and showed a significantly positive effect on CMJ performance (Tomljanović et al., 2011). After an 8-week intervention program, the High intensity interval training (HIIT) with functional exercises showed no significant differences in CMJ (Alonso-Fernández et al., 2017). However, the CG did not show significant changes in any studied variables. One study for Squat jump demonstrated that traditional strength training group considerably improved performance when compared to FT, jump training, and sprint training groups (Keiner et al., 2020). Functional resistance training with elastic bands had a statistically significant advantage in improving quadrant jump test and differences between before and after test of assessments was significant at the 0.05 significance level (Abdel-Aziz Habib, 2018).

### Effect of FT on functional movements

FMS performance was measured in four studies involving five experimental groups (Yildiz et al., 2019; Baron et al., 2020; Kovac et al., 2022; Li, 2022). There was no difference between CG and TTG when the FMS data were considered (*p* > 0.05). However, significant variations appeared between CG and FTG as well as between TTG and FTG on FMS (*p* = 0.001) (Yildiz et al., 2019). In one study, the intervention group’s FMS score was considerably higher after the 6-week training intervention (f = 9.85, *p* = 0.004). Nonetheless, considering that individual.

Assessments showed comparable group and time effects, it is possible to attribute the variations in total score primarily to group differences in the trunk stability push-up (*p* = 0.02) and active straight leg raise assessments (*p* = 0.004) (Kovac et al., 2022). The functional ability of young football players has also significantly improved according to the FMS results—deep squat (45.2% of difference, *p* = 0.004), hurdle step (24.3% of difference, *p* = 0.012), in-line lunge (48.5% of difference, *p* = 0.001) (Baron et al., 2020). On the other hand, the study presented that the average score of the seven items of functional action screening before and after the experiment increased from 13 to 14.77 points, with a *p*-value less than 0.01. The result shows that FT effectively improves the FMS level of middle school football team players (Li, 2022).

## Discussion

The main objective of this review is to illuminate the effects of functional training on athletes’ capacities for sprinting, jumping, and performing functional movements. Sprinting, jumping, and functional movements were utilized as outcome measures to effectively evaluate the advantages of FT interventions vis-à-vis the control or alternative training methods. The major finding of this systematic review is that FT interventions improved athletes’ performance on tests measured from their propensity to sprint, jump, and their use functional movement skills. The results of the current review studies are, however, being updated and supplemented with respect to the effects of FT treatments on jumping performance and FMS in the athletic population. Improvements in FMS, sprinting, and jumping performance were seen in each study that was included in this systematic review. However, there were some differences between the research that studied the FT interventions in terms of the frequency of training sessions conducted per week, the overall length of the training interventions, the length of the intervention period, and the outcome measures. The examined studies also vary considerably in terms of the characteristics of participants—athletes, age, and gender—and the physical fitness components being assessed. Given the mostly positive outcomes of these research, FT may be a helpful strategy for athletes. Functional training was found to be effective for young athletes who play team sports in their 5, 10, 15, 20, 25, and 30 m sprinting performances (Sander et al., 2013; Bhardwaj and Kathayat, 2021). As a side note, only a few studies were conducted on female and mixed-gender athletes (male and female). There is also few research on elite, moderately trained, and university athletes.

In comparing training groups, elite adolescent soccer players’ 20-m linear sprint and squat jump performance changes over 10 months of plyometric, sprint, functional, and traditional strength training were examined. According to this study, the traditional strength training (TST) group outperformed the sprint and jump training as well as FT groups in terms of speed and squat jump parameters (Keiner et al., 2020). Based on the results, research indicates that the FT (mini band and body mass exercises, 2-5 sets with 10 repetitions and a rest of 2 min between sets, each) employed in this study cannot be recommended for increasing maximum strength or enhancing sprinting and leaping abilities (Keiner et al., 2020). Meanwhile, scientific studies conducted on the impacts of applying 8 weeks of FT training with the frequency of 5 days per week and 60 min session duration show positive effects on 30-m sprinting and vertical jumping in comparison to the traditional training intervention group that are statistically significant. Additionally, compared to CG, the improvement was shown in both the TTG and FTG. There were substantial variations between all performance values of the groups at the conclusion of the 8-week training period. When compared to CG, TTG’s vertical jump values showed a significant improvement (*p* < 0.05). With 50%–70% intensity of FTG demonstrated considerable improvements in all performance metrics as compared to CG. These improvements were found to be normal when the fitness-training programs—TT and FT—were considered. When comparing FTG with TTG, the former significantly improved on every other performance measure due to FT. On top of that, there was no discernible difference between CG and TTG when FMS data were analyzed (*p* > 0.05), however, there was a difference between CG and FTG and between TTG and FTG (*p*, 0.001) (Yildiz et al., 2019). On the other hand, could not find a significant difference between 5-week FT and TT exercises (Tomljanović et al., 2011).

The effects of high intensity FT (HIFT) on participants’ athletic fitness with different training loads and frequencies were assessed using baseline measurements of 20 m sprinting performance and CMJ height at 6 weeks. There are two groups: High training volume and frequency (HTVF) and moderate training volume and frequency (MTVF). The results showed that neither groups’ performance in the countermovement vertical jump or running could be improved by 6 weeks of high-intensity FT. Therefore, it appears that the improvement in the physical performance of HIFT practitioners is unaffected by different volumes and frequencies (Teixeira et al., 2020). 121 professional youth soccer players between the ages of 13–18 took part in this study and completed two warm-up programs. They were separated into two parts and conducted the usual warm-up for soccer (NWP) training group and the same warm-up with functional exercises (WPS) training group. The findings demonstrate that, after 8 days of training, adding functional activities to a general warm-up had no impact on sprint performance (Sander et al., 2013). Researchers return this to the effectiveness of the 10-week with 120 min training duration of functional resistance drills with elastic bands applied during the specific preparation phase of the training program for junior soccer players. According to the study, there are statistically significant variations in the mean measurements of the study sample’s pre- and post-tests, with the level test (0.05) in the quadrant jumping test (Abdel-Aziz Habib, 2018). The study demonstrates that a 6-week corrective exercise program improved the overall FMS score of female netball players. Shoulder mobility, in-line lunge, deep squat, and rotary stability may not have affected the hurdle step scores of the intervention groups. Although the level of achievement is equivalent to that in other studies, population sample, intervention mode, and intervention duration differences should be properly taken into account when comparing results (Kovac et al., 2022). Moreover, the highest overall FMS score is 21 points, with passing score of 14. Subjects with a score below 14. The failing rate increased to 14.77 points and exceeded the passing line with *p*-value less than 0.05. This shows that the before and after test results had significant differences. It also shows that the students’ FMS level has significantly improved through the practical training intervention for 8 weeks, 2 days a week (Li, 2022). Furthermore, after 8 weeks training intervention also have a positive result on FMS in comparison to traditional training group and control group. According to the study, FT is different from traditional training in the sense that it may involve exercises carried out to improve a particular movement (Pacheco et al., 2013).

Regarding intervention duration, frequency, and FT sessions length, this systematic review found that limited training factors predict FT effects on the squat jump, quadrant jump, 5-m sprint, 15-m sprint, 25-m sprint, 50-m sprint performance, and FMS in athletes. However, reviews for intervention duration, frequency, and FT sessions length per week were available for sprinting, jumping, and functional movement performance in athletes. Interventions that were conducted for a duration of at least 8 weeks, with a frequency of three sessions per week and at least 60 min FT session duration induced a more significant beneficial training effect on 10-m sprint, 20-m sprint, 30-m sprint, jumping performance, and functional movement skills, compared to those interventions with less than 8 weeks, with frequency less than three sessions per week and shorter than 60 min FT session duration. Likewise, some studies reported no change after intervention in the vertical jump (Elbadry, 2014; Teixeira et al., 2020), countermovement jump (Alonso-Fernández et al., 2017), squat jump (Keiner et al., 2020), five- meter sprint (Tomljanović et al., 2011; Baron et al., 2020), 10-m sprint (Tomljanović et al., 2011; Yildiz et al., 2019), 20-m sprint performance (Tomljanović et al., 2011; Keiner et al., 2020; Teixeira et al., 2020) and FMS (Yildiz et al., 2019).

The outcome of this review demonstrates that sprinting, jumping, and FMS status after the training intervention has improved significantly. Several studies suggested that FMS is essentially an evaluation tool that coaches and trainers can use to create a program which includes functional exercises designed for various athletes (Yildiz et al., 2019; Baron et al., 2020; Kovac et al., 2022; Li, 2022). The studies proposed that FT can be a great technique to enhance training at various points of the macrocycle if it is intended to shape fundamental motor skills or remove functional restrictions (pre-hab). Based on existing insights (Baniasadi et al., 2019; Abdolahi et al., 2020; Molavi-Taleghani et al., 2020) and the results shown here urges the practitioners and coaches into thinking about requesting admission for their athletes into a longer training course (Baniasadi et al., 2019; Sheikhbardsiri et al., 2019; Sheikhbardsiri et al., 2020). The study’s practical application is that using FMS requirement specification can help and strength and conditioning coaches identify weaker movement patterns and improve them, effectively improving their overall training program. Players may be protected from harm and have their speed characteristics improved by implementing the FT program in a regular micro-cycle (Baron et al., 2020). Moreover, 5-m sprint, 10-m sprint, 15-m sprint, 20-m sprint, 25-m sprint, 30-m sprint, 50-m sprint performance, countermovement jump, vertical jump, quadrant jump, and squat jump may be improved significantly. Some studies did not find positive effects after training intervention in the 5-m sprint, 20-m sprint, vertical jump, squat jump, and countermovement jump due to additional exercises, short duration of intervention, and frequency (Elbadry, 2014; Alonso-Fernández et al., 2017; Baron et al., 2020; Keiner et al., 2020; Teixeira et al., 2020).

According to the conceptual study that there is no specific definition of FT. However, the FT program and exercises are similar to other types of training (Ide et al., 2022). On the other hand, there is strong evidence that FT is different from other types of training and that it has a positive influence on athletes’ performance (Liu et al., 2014; McLaughlin et al., 2020; Wilke and Mohr, 2020; Chen et al., 2021; Xiao et al., 2021). In functional training, lower and upper body movements involve muscle and joint movements (Brill, 2008). This concept supports the idea of functional exercise, which consists of exercises based on the movement that will apply in contrast to traditional training, which is focused on the development of muscles in an isolated manner. In another study, functional training is advantageous because all the natural movements involve multiple joints and different motion planes rather than isolation (Turna and Alp, 2020). Furthermore, functional training has recently gained popularity in physical fitness training despite being a relatively new training method. It has been named one of the “Top 10 Fitness Trends” for 2018 (Thompson, 2017), with eight of the fifteen studies published in the past 3 years.

## Strengths and limitations

There are several strengths to this systematic review. The PRISMA statements served as the review’s guiding principles, and the PEDro scale was used the risk of bias in each included study. Three independent and blinded evaluators also carried out screening and bias evaluations. Finally, all of the included articles were randomized controlled trials.

However, there are a lot of limitations on the current review. The first limitation is that there are limited existing studies on the squat jump, quadrant jump, and functional movement skills. Secondly, the reviewed studies mostly use short FT intervention durations (Tomljanović et al., 2011; Sander et al., 2013; Alonso-Fernández et al., 2017; Teixeira et al., 2020; Bhardwaj and Kathayat, 2021; Kovac et al., 2022). Thirdly, most of the studies did not report about intensity of the intervention program except two. Additionally, most of the included studies showed a rather small sample size per group (Tomljanović et al., 2011; Elbadry, 2014; Alonso-Fernández et al., 2017; Yildiz et al., 2019; Keiner et al., 2020; Turna and Alp, 2020; Usgu et al., 2020; Li, 2022). On top of that, the studies did not mention the sample size calculation method for choosing these athletes. Lastly, there is limited studies on elite youth athletes, moderately trained athletes, college-level athletes, as well as female and mixed-gender (male and female) athletes.

## Conclusions and recommendations

The main conclusion from this review is FT interventions group in comparison to control group are likely to increase athletes’ performance. Athletes’ sprinting, jumping, and functional movement skills improved due to FT. This is despite the total duration of the training interventions differing across studies included in this systematic review. As a result of the analysis, it appears that training time, frequency, and intensity are significant factors to consider when developing an FT intervention for athletes. On the other hand, more research is needed to determine the impact of various FT periods on athletes’ sprinting, jumping, and functional mobility. Furthermore, FT should also be applied more frequently in other sports (hockey, handball, volleyball, cricket, badminton, and athletics) to allow its benefits on performance and movement abilities in all levels of male and female players to be observed.

## Practical application

Faster directions influenced players’ speed, jump, and movement abilities, which were necessary for player performance. Considering the growing focus on functional training, it is crucial for researchers to assess and contrast the efficacy and long-term effects of these programs on athletes’ performance. Based on current available evidence, functional training is helpful to enhance the speed, jumping, functional movements, and physical fitness components among various types of athletes (Osipov et al., 2017). Moreover, reviewed trials show a great difference in research design, participant recruitment criteria, and functional training programs. We identified three patterns of functional training: element-based functional training, task-specific- based functional training, and hybrid functional training. Accordingly, coaches should do functional training sessions lasting 60 min, three times a week, for 12 weeks. All athletes participating in this program appear to gain from increased speed, leap, and functional movements.

## Data Availability

The original contributions presented in the study are included in the article/supplementary materials, further inquiries can be directed to the corresponding authors.
